# Utility of the ISTH bleeding assessment tool (BAT) in diagnosis of Glanzmann Thrombasthenia patients

**DOI:** 10.12669/pjms.38.4.5361

**Published:** 2022

**Authors:** Nazish Saqlain, Tooba Fateen, Hammad Tufail, Naghmana Mazher

**Affiliations:** 1 Dr. Nazish Saqlain, FCPS (Hematology), Department of Hematology and Transfusion Medicine, The Children’s Hospital & Institute of Child Health, Lahore, Pakistan; 2 Dr. Tooba Fateen, FCPS (Hematology), Department of Hematology and Transfusion Medicine, The Children’s Hospital & Institute of Child Health, Lahore, Pakistan; 3 Dr. Hammad Tufail Chaudhary, FCPS (Hematology). Hematology Department, Taif University, Taif KSA; 4 Dr. Naghmana Mazher, M. Phil (Hematology). Pathology Department, Fatima Jinnah Medical University, Lahore, Pakistan

**Keywords:** Glanzmann Thrombasthenia, Platelet function disorders, Bleeding, Children, Questionnaire

## Abstract

**Objectives:**

To assess the utility of ISTH-BAT (International Society on Thrombosis and Hemostasis- Bleeding Assessment Tool) in the diagnosis of Glanzmann Thrombasthenia (GT) in comparison to controls.

**Methods:**

It was a case-control study carried out at The Children’s Hospital, Lahore from January 2012 to May 2021. All patients from neonates to 18 years with a final diagnosis of GT were studied retrospectively. The clinical details were collected from hospital records and telephonically on ISTH-BAT questionnaire after taking informed consent. The same proforma was obtained from 75 healthy controls. Data was analyzed on SPSS version 26.

**Results:**

Out of 427 patients with suspected platelet function disorders, 133 were diagnosed as GT. The mean age was 7.29±5 years. Male to female ratio was 1.1:1. Among cases, 76.6% were products of consanguineous marriage. Epistaxis was the commonest symptom with highest score (p value<0.001). Cutaneous and oral cavity bleeds were more severe and frequent in patients than controls (p value < 0.004). The median ISTH-BAT score among patients was nine while in control group was one. Sensitivity was 86.4%, specificity was 77.3%, positive predictive value was 0.87 and negative predictive value was 0.76. Area under the receiver operator curve was 0.78 (95% confidence interval 0.82–0.90, p< 0.001*).

**Conclusion:**

ISTH-BAT scores were significantly higher in GT patients than controls. So, we recommend the inclusion of ISTH-BAT in diagnostic evaluation of patients with suspected Glanzmann Thrombasthenia.

## INTRODUCTION

One of the most common hereditary bleeding disorders is Platelet function disorders (PFDs). These patients can undergo bleeding after trauma or sometimes spontaneously in case of severity.^1^ In 1918, a rare mucocutaneous bleeding disorder was identified by Eduard Glanzmann, and it was named after the scientist as Glanzmann thrombasthenia (GT). The range of intensity of signs and symptoms of this disease is variable. Usually, it is diagnosed early in life due to symptoms like petechiae, purpura and easy bruising. Menorrhagia at childbearing age, epistaxis, bleeding from gums are quite common symptoms in this disease. Hematuria, bleeding from gastrointestinal tract and intracranial hemorrhage is seen less common. Frequently, platelet size and number remain normal.^2^

Diagnosis of these patients of GT is not an easy task. Diagnosis is based on different kind of complicated laboratory assays like light transmission aggregometry.^3^ These laboratory tests need extensive amount of cost, hard work and complexity in interpretation. Therefore, specialized laboratories are needed which are only placed in specialized centers.^4^,^5^ These centers are present in big cities which lead to lack of access of major part of population.

Therefore, structured bleeding assessment tools (BATs) can be helpful to screen the suspected patients. BAT from the International Society on Thrombosis and Hemostasis (ISTH-BAT) is one of the well-known tools for this purpose. The ISTH-BAT is a diagnostic questionnaire to screen patient depending on presence and absence of bleeding and also their severity.^6^ This method has been extensively validated for von Willebrand disease (VWD).^7^ Therefore, we aimed to test this method for our group of patients. As it might help to screen the patients before detailed investigations which can help to use the resources adequately. The objective of the study was to assess the diagnostic utility of ISTH-BAT in the diagnosis of Glanzmann’s thrombasthenia patients by comparing it with healthy controls.

## METHODS

We included all patients from neonates to 18 years of age referred with a suspected platelet function disorder to The Children’s Hospital and Institute of Child Health, Lahore, Pakistan from January 2012 to May 2021. Informed consent was obtained from parents/guardians of the participants telephonically after approval of the study protocol by Institutional ethics committee [IRB# 2020-184-CHICH]. Patients who refused to participate in the study and those with incomplete history were excluded. ISTH-BAT proforma was used for evaluation of bleeding history of each individual obtained from the records (Annexure I). According to this questionnaire each bleeding symptom was assigned a score (0–4) depending on the severity of symptoms. Post-partum bleeding was excluded from the proforma as the participants were in pediatric age group.

The laboratory tests results taken into account were bleeding time and Platelet aggregation studies. Bleeding time was performed by Ivy’s method as per laboratory protocol and considered to be prolonged when it came more than nine minutes. Platelet aggregation study results were noted to select cases. It was done by collection of blood into evacuated tubes containing 3.2% sodium citrate and EDTA tube for platelet count and morphology. Platelet function assessment was done with light transmission aggregometry (LTA) within 2 hours of sample collection by preparing Platelet-rich plasma (PRP). PRP was prepared from whole blood centrifugation at 170–200 g for 10 minutes. Before proceeding platelet, count was checked to be in the range of 200–300x 10^9^/L.

Platelet aggregation test was prepared on 133 patients, response was measured with ADP (10 mM), epinephrine (10 mM), Ristocetin (1.25 mg/mL) and collagen (2 mg/mL). Results were classified as abnormal when aggregation is found to be less than 50% for each agonist. The diagnosis of Glanzmann Thrombasthenia (GT) was made when aggregation was present only with Ristocetin and absent with other agonists.

Same ISTH-BAT proforma was filled from 75 healthy controls after their informed consent, followed by Platelet aggregation studies by the same protocol as in cases. Data was analyzed through SPSS version 26. Qualitative variables like gender and bleeding presentation are represented as frequency and percentage. ISTH bleeding score are presented as median. Sensitivity, specificity, positive and negative predictive values were calculated for the proforma.

The data presented at ISTH- congress 2020 has been revised with change of time period, as few ambiguous cases have been removed and replaced with new ones.^8^

## RESULTS

Out of 427 patients who presented with suspected platelet function disorders, 133 were diagnosed as Glanzmann Thrombasthenia on LTA. The mean age of GT patients was 7.29 years (± 5 SD), and healthy volunteer controls was 21 years (± 10 SD). Male to female ratio in cases and control group was 1.1:1 and 1:1 respectively. About 76.6% patients were born to parents with consanguineous marriages. History of bleeding in first degree relatives was present in 62.6% patients (p<0.001^*^) and while sibling death due to bleeding was found in 11 patients of GT. In some cases, sibling death was reported but the cause was not established so those were excluded. All the controls had normal results on platelet aggregation studies.

The subgroup analysis of different signs and symptoms in terms of bleeding severity showed that Epistaxis (p= 0.001^*^) and cutaneous wound bleeding (p= 0.004^*^) symptoms were significantly severe and frequent in case as compared to controls. (Table-I)

**Table-I:** ISTH- BAT score according to bleeding symptoms in cases and controls.

	Cases Median (Interquartile Range)	Control Median (Interquartile Range)	p-value
Epistaxis	3 (2-4)	2 (1-2)	0.001*Significant
Cutaneous/Wounds	2 (1-2)	1 (0-1)	0.004* Significant
Oral/Gums	2 (1-2)	2 (0-2)	1.00
GIT	1 (1-4)	0	
Tooth	4 (1-4)	0	
Surgery	4 (2-4)	0	
Menorrhagia	1 (1-1)	1	0.536
Hematoma	2 (1-2)	0	
Hemarthrosis	1 (0)	0	
CNS bleed	2 (0-1)	0	
Others (circumcision/ cephalohematoma/ umbilical stump)	4 (1-4)	0	

ISTH-BAT Proforma found to have a sensitivity of 86.4%, specificity of 77.3%, positive predictive value of 0.87 and Negative predictive value of 0.76 in terms of evaluation and diagnosis of GT patients. This revealed the high screening reliability of this tool. (Table-II)

**Table-II:** Sensitivity, Specificity, Positive Predictive and Negative predictive value of ISTH-BAT in diagnosing GT.

SENSITIVITY	86.4%
Specificity	77.3%
Positive Predictive Value	0.87
Negative Predictive Value	0.76

**Fig.1:**
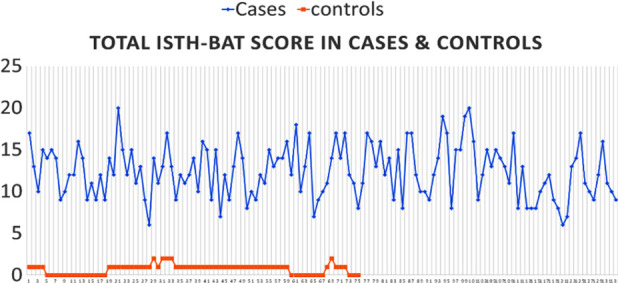
Total ITH-BAT Score in Cases & Controls

The median ISTH-BAT score in GT patients was nine while in control group it was one. 84.2% GT patients had a score of ≥7, falling into more severe bleeding category. (Fig.1)

**Fig.2:**
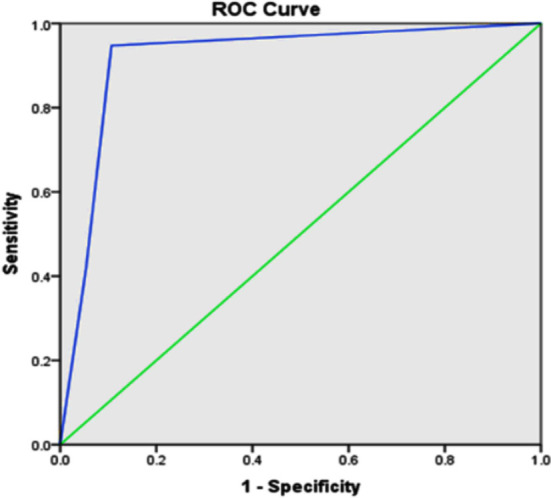
Receiver Operating curve (ROC) for the presence of GT and the ISTH-BAT score.

A ROC curve analysis has shown that ISTH-BAT can reliably differentiate between patients with and without Glanzmann Thrombasthenia having an area under the curve of 0.78 (95% confidence interval 0.82–0.90, p< 0.001^*^) (Fig.2).

## DISCUSSION

In our study, we found that 133 patients out of 427 suspected bleeding disorder patients were diagnosed as GT by LTA. It constitutes 31.1 % (133 out of 427) of cases. It is little higher than other studies in which 20.4 % patients (33 out of 161) were diagnosed as GT. The reason for this difference might be that sample size in other study is much less than our study.^9^

The mean age at presentation was 7.29 years ± 5. This is almost same as found in other studies from Pakistan.^9^,^10^ Again 76.6% patients were born in families with consanguineous marriage. Same thing has been mentioned in other studies that consanguineous marriages are common in Pakistan, which can lead to persistence of genetic disease in family.^3^

Sensitivity of this method was 86.4 % while specificity was 77.3%. Positive predictive value is 0.87 and negative predictive value is 0.76. This is in line with other studies done on similar topic to assess the reliability of ISTH-BAT.^11^ The questionnaire can be a beneficial tool in screening patients with bleeding disorders including those with coagulation and/or platelet function defects but it cannot pinpoint the illness.

Median bleeding score in control group was one. This is same as shown in other studies like that is done by Rashid A et al.^9^ Median bleeding score for cases was nine. This is significantly different from control group. Alder et al. study showed high bleeding median scores in pediatric population with platelet function defect, as in our study.^11^ Same kind of observations were mentioned in another recent study by Gresele P.^12^ The high bleeding score may be due to the existence of more severe types of GT in our population or late presentation to the hospital. In a study done on Pakistani population, suspected PFD were compared to control in terms of ISTH-BAT and found significantly different.^9^ Similar study by Lowe et al. showed significant difference in scores of ISTH-BAT when compared between group of excessive bleeding patient and healthy volunteers.^13^ Parez et al. and Kaur et al. also compared bleeding disorders with normal controls in terms of ISTH-BAT and found them significantly different.^14^,^15^

In our patient group, family history of bleeding in first degree relatives was present in 62.6% patients which has been significantly different from cases (p<0.001*). History of sibling death due to bleeding was found in 11 patients of GT. So, a meticulous family history of bleeding is found to be important in high suspicion of an inherited bleeding disorder including GT. Similar findings were reported by Rashid A et al.^9^

If we assess the bleeding scores of different signs and symptoms, we can see that epistaxis and cutaneous wound bleeding symptoms were found significantly different from control group. Other study didn’t show such amount of significance although platelet function defect group showed epistaxis and cutaneous wound bleeding more than control group. The difference from our study might be due to the reason that their study had less patient and bleeding symptoms were considered for all patients of platelet function defects in contrast to our study in which only GT is considered.^16^ This finding is in favor of inference that Epistaxis and cutaneous wound bleeding symptom are more important to diagnose GT. Oral gum bleeding had no significant difference in both control and case groups. Same finding was found in a similar study.^17^

All other symptoms like tooth extraction bleeding, bleeding during surgery, hematoma, hemarthrosis and CNS bleeding were only found in GT patients’ group. No case was there in control group. Similar findings were found in other study.^18^ It showed that these are very important signs or symptoms to include platelet function disorders in differential.

### Strength and Limitations of the study:

Our study was unique in a sense that it focused only on GT in contrast to other studies which usually included many PFD. To gather such number of patients of rare disease was demanding. The limitations of our study were not including flowcytometry and molecular analysis for GT. Different mutations leading to the disease can present in a different way.

Our results favor the importance of the use of ISTH-BAT scoring in suspicious patients of GT. High sensitivity and predictive values favor our statement as in other studies.^11^,^19^ This use of ISTH-BAT becomes more important in 3^rd^ world countries with low capita income and also low health budget of governments. Scarcity of Tertiary care hospitals who are equipped with diagnostic tools leads to underdiagnosis and more morbidity and mortality in patients of platelet function defects. Application of ISTH-BAT will help to screen the patients and to refer only selective patients to the tertiary care hospitals which will help to manage the budget and resources adequately.

## CONCLUSION

Our study has been based on a large number of pediatric GT patients with more severe clinical presentations. ISTH-BAT scores were significantly high in GT patients as compared to controls. We recommend the inclusion of ISTH bleeding assessment tools questionnaire for screening of patients with a suspected platelet function disorder including GT before proceeding to laborious investigations.

### Authors’ Contribution:

**NS:** Conceived, designed did statistical analysis & editing of manuscript, responsible and accountable for the accuracy or integrity of the work.

**TF, HT:** Did data collection and manuscript writing.

**NM:** Did review and final approval of manuscript.
